# Potentially Life-Threatening Interaction between Opioids and Intrathecal Baclofen in Individuals with a Childhood-Onset Neurological Disorder: A Case Series and Review of the Literature

**DOI:** 10.1055/s-0044-1787103

**Published:** 2024-05-22

**Authors:** Liza M.M. van Dijk, Annelies van Zwol, Annemieke I. Buizer, Laura A. van de Pol, K. Mariam Slot, Saskia N. de Wildt, Laura A. Bonouvrié

**Affiliations:** 1 Department of Rehabilitation Medicine, Amsterdam University Medical Centers, Location Vrije Universiteit, Amsterdam, the Netherlands; 2 Amsterdam Movement Sciences, Rehabilitation & Development, Amsterdam, The Netherlands; 3 Department of Intensive Care Medicine, Radboud University Medical Center, Nijmegen, The Netherlands; 4 Emma Children's Hospital, Amsterdam University Medical Centers, Location University of Amsterdam, Amsterdam, The Netherlands; 5 Department of Child Neurology, Amsterdam University Medical Centers, Location Vrije Universiteit, Amsterdam, The Netherlands; 6 Department of Neurosurgery, Amsterdam University Medical Centers, Location University of Amsterdam, Amsterdam, The Netherlands; 7 Brain Tumor Center Amsterdam, Amsterdam University Medical Centers, Amsterdam, The Netherlands; 8 Division of Pharmacology and Toxicology, Department of Pharmacy, Radboud University Medical Center, Nijmegen, The Netherlands; 9 Department of Neonatal and Pediatric Intensive Care, Erasmus MC-Sophia Children's Hospital, Rotterdam, The Netherlands

**Keywords:** baclofen, opioids, respiratory depression, decreased consciousness, drug interaction

## Abstract

**Background** Spasticity and dystonia are movement impairments that can occur in childhood-onset neurological disorders. Severely affected individuals can be treated with intrathecal baclofen (ITB). Concomitant use of ITB and opioids has been associated with central nervous system (CNS) depression. This study aims to describe the clinical management of this interaction, based on a case series and review of literature.

**Methods** Four individuals with childhood-onset CNS disorders (age 8–24) and CNS-depressant overdose symptoms after the concomitant use of ITB and opioids are described. The Drug Interaction Probability Scale (DIPS) was calculated to assess the cause-relationship (doubtful <2, possible 2–4, probable 5–8, and highly probable >8) of the potential drug–drug interaction. A literature review of similar previously reported cases and the possible pharmacological mechanisms of opioid–baclofen interaction is provided.

**Results** After ITB and opioid co-administration, three out of four patients had decreased consciousness, and three developed respiratory depression. DIPS scores indicated a possible cause-relationship in one patient (DIPS: 4) and a probable cause-relationship in the others (DIPS: 6, 6, and 8). Discontinuation or adjusting ITB or opioid dosages resulted in clinical recovery. All patients recovered completely. In the literature, two articles describing nine unique cases were found.

**Conclusion** Although the opioid–ITB interaction is incompletely understood, concomitant use may enhance the risk of symptoms of CNS-depressant overdose, which are potentially life-threatening. If concomitant use is desirable, we strongly recommend to closely monitor these patients to detect interaction symptoms early. Awareness and monitoring of the potential opioid–ITB interaction is essential to reduce the risk of severe complications.

## Introduction

Spasticity and dystonia are movement impairments that may occur in various childhood-onset neurological disorders, such as cerebral palsy or neurogenetic diseases. 1 2 3 4 These impairments are often a target of treatment, as they can cause problems with comfort, mobility, communication, and personal care and negatively affect quality of life of the patients as well as their caregivers. Frequently, oral pharmacological therapy is the first step in medical treatment, but this is often not sufficient in severely affected individuals and side-effects are common. In these individuals, intrathecal baclofen (ITB) therapy can be considered. 5

Baclofen, a gamma-aminobutyric acid B (GABA _B_ ) agonist, is a centrally acting muscle relaxant which has central nervous system (CNS)-depressant properties. It decreases spasticity by inhibiting neuronal transmission in the lower (predominantly) alpha motor neurons at the spinal level. 6 7 It can also reduce dystonia, presumably by suppressing the excessive stimulation of the supplementary motor and premotor cortex at the cerebral level. 6 8 As the site of action is the CNS, baclofen must cross the blood–brain barrier (BBB) or blood–cerebrospinal fluid barrier (BCSFB) when administered systemically. Since baclofen is poorly lipid soluble, it passes these barriers poorly, and high plasma concentrations are needed to reach sufficient cerebrospinal fluid (CSF) concentrations. 9 These high plasma concentrations may lead to unwanted side effects such as nausea, drowsiness, and sedation, while a sufficient therapeutic response may be lacking. 10 By administering baclofen intrathecally using an implanted microinfusion pump, this problem can be bypassed. 11 The pump is implanted in the abdominal wall and connected to the intrathecal space via a catheter, providing a continuous infusion of baclofen, which allows sufficient CFS concentration levels to be achieved while keeping the plasma concentration level low. 12

Individuals on ITB therapy often have significant comorbidities, such as severe scoliosis, that require surgery. 13 After any type of surgery, including baclofen pump implantation, pain prevention is an important starting point in managing postoperative pain since the goal is to wake up from surgery with sufficient pain control. 14 Properly functioning analgesia is of great importance in individuals with an ITB pump, as discomfort can trigger or exacerbate spasticity and dystonia. 15 In moderate or severe pain, opioids are widely used. 14 16 For optimal analgesic efficacy, these opioids must reach effective concentrations in the CNS. 16 Opioids have a relatively favorable ability to cross the BBB due to their higher lipophilicity. 17 However, concurrently using opioids and skeletal muscle relaxants (SMRs), such as baclofen, may increase the risk of symptoms of CNS-depressant overdose, such as sedation and respiratory depression, due to a possible synergistic effect. 18 19 20 21

There is limited information in the literature on the clinical consequences of an interaction effect between opioids and ITB, and health care professionals may not always be aware of this interaction, which can be potentially life-threatening. In this study, we describe four individuals with a childhood-onset neurological disorder who suffered serious symptoms of CNS depression after concomitant use of opioids and ITB. We review the literature for previously described cases and provide suggestions for preventing the interaction and its clinical management.

## Methods

This article presents four cases with symptoms of CNS-depressant overdose after concomitant use of ITB and opioids that occurred in the Amsterdam University Medical Center (UMC) and Radboud UMC between 2010 and 2022. According to the Research Ethics Board of Amsterdam UMC, formal approval is not required for this study (#2022.0883). The guardians of two patients provided informed consent to use their child's data. At the time of this study, the other two patients presented were deceased, not due to complications from the coadministration of opioids and ITB. We did not ask for consent from their relatives because we considered it too burdensome, and patient data were anonymized. This is according to the Dutch Code of Conduct for Health Research.

Data were collected through medical record screening. The Drug Interaction Probability Scale (DIPS), which consists of 10 questions, was then calculated for each patient to assess a possible causal relationship of a potential drug–drug interaction between opioids and ITB and the clinical sign. 22 23 A score less than 2 indicates a doubtful cause relationship, a score between 2 and 4 indicates a possible cause relationship, a score between 5 and 8 indicates a probable cause relationship, and a score greater than 8 indicates a high probable cause relationship. 22

The literature search was conducted using PubMed, incorporating the search terms: baclofen, ITB, SMRs, morphine, opioids, drug interactions, adverse reactions, and side effects. A snowballing approach was used to identify potentially relevant articles that were not identified in the initial search.

## Results

Patient characteristics and calculated DIPS scores of the four described cases are presented in Tables 1 and 2 .

**Table 1:** Patient characteristics of the four described cases

	Case 1	Case 2	Case 3	Case 4
Year of interaction	2010	2012	2021	2022
Sex/age (y)	F/8	M/24	M/14	F/15
Weight (kg)	22	44	60	34.5
Diagnoses	Bilateral SCP, GMFCS level V, due to porencephalic cyst with hydrocephalus and ventriculoperitoneal shunt	Neurodegenerative disorder of unknown cause, with bilateral spasticity	Neurodegeneration with brain iron accumulation due to compound heterozygous *PKAN2* mutations, with dystonia	Bilateral SCP, GMFCS level V due to microcephaly
Medical history	PEG-tube; epilepsy	PEG-tube; ventilation impairment due to severe scoliosis		Epilepsy; recurrent gastroparesis with nausea and vomiting; severe OSAS; PEG-tube
Medication at the time of admission	Valproic acid (200 mg, 2 dd)	ITB (11 y, 672 µg/d); omeprazole (40 mg, 2 dd); calcium carbonate/colecalciferole (500 mg, 2 dd); bisacodyl (10 mg, 3/wk); macrogol	ITB (7 mo, 365.5 µg/d); baclofen (orally, 5 mg); clonazepam (1.5 mg); gabapentin (600 mg)	ITB (4 y, 507 mcg/d); baclofen (orally, 5 mg 3 dd when needed); valproic acid (500 mg, 2 dd); perampanel (4 mg 1 dd); aprepitant (80 mg 1 dd); omeprazole (20 mg, 2 dd); glycopyrronium (1,280 µg, 2 dd)

Abbreviations: dd, daily; F, female; GMFCS, gross motor function classification system; ITB, intrathecal baclofen; OSAS, obstructive sleep apnea syndrome; PEG, percutaneous endoscopic gastrostomy; PKAN, pantothenate kinase-associated neurodegeneration; SCP, spastic cerebral palsy.

**Table 2:** Calculated Drug Interaction Probability Scale scores the four described cases

Item	Score per case			
	1	2	3	4
1. Are there previous credible reports of this interaction in humans? (yes +1; no −1; Unk/NA 0)	0	0	0	0
2. Is the observed interaction consistent with the known interactive properties of the precipitant drug? (yes +1; no −1; Unk/NA 0)	+1	+1	+1	+1
3. Is the observed interaction consistent with the known interactive properties of the object drug? (yes +1; no −1; Unk/NA 0)	+1	+1	+1	+1
4. Is the event consistent with the known or reasonable time course of the interaction (onset and/or offset)? (yes +1; no −1; Unk/NA 0)	+1	0	+1	+1
5. Did the interaction remit upon the dechallenge of the precipitant drug with no change in the object drug? (if no dechallenge, use unknown or not applicable and skip question 6) (yes +1; no −2; Unk/NA 0)	+1	+1	+1	+1
6. Did the interaction reappear when the precipitant drug was readministered in the presence of continued use of object drug? (yes +2; no −1; Unk/NA 0)	0	0	+2	0
7. Are there reasonable alternative causes for the event? (yes −1; no +1; Unk/NA 0)	0	−1	0	0
8. Was the object drug detected in the blood or other fluids in concentrations consistent with the proposed interaction? (yes +1; no 0; Unk/NA 0)	0	0	0	0
9. Was the drug interaction confirmed by any objective evidence consistent with the effects on the object drug (other than drug concentrations from question 8)? (yes +1; no 0; Unk/NA 0)	+1	+1	+1	+1
10. Was the interaction greater when the precipitant drug dose was increased or less when the precipitant dose was decreased? (yes +1; no −1; Unk/NA 0)	+1	+1	+1	+1
**Total score**	6	4	8	6

Abbreviations: NA, not applicable; Unk, unknown.

Note: Total score >8: highly probable; 5–8: probable; 2–4: possible; <2: doubtful. For guidelines on how to use the DIPS, see the paper by Horn et al. 22

### Case 1

An 8-year-old girl with bilateral spastic cerebral palsy and epilepsy was hospitalized for baclofen pump implantation. Perioperatively, she received three doses of fentanyl of 50, 25, and 25 mcg intravenously (i.v.) (concentration 50 mcg/mL). After surgery, she received 7 mg of piritramide (i.v.) at the postanesthesia care unit (PACU) to achieve adequate pain relief. There were no complications during surgery or at the PACU, and she was transferred to the pediatric ward. In the late afternoon, the baclofen pump was started and set at a dosage of 50 mcg/d. At that time, the patient was lucid and normally responsive, according to her parents. Three hours later, she was unresponsive to a pain stimulus and hypotonic, at which the baclofen infusion was lowered to 12 mcg/d. One hour later, she still had a decreased consciousness, but did open her eyes occasionally. Her muscle tone had somewhat increased. An electroencephalogram (EEG) ruled out a nonconvulsive status epilepticus as a cause for the decreased consciousness. The EEG showed an encephalopathy characterized by asymmetric, right more than left, slowing of the background pattern with frequent interictal epileptiform discharges. Over the next 2 days, her consciousness improved slowly, and 2 days after the baclofen therapy was started, her consciousness was normal again. Four days later, the patient was discharged with the baclofen pump set at 12 mcg/d. The baclofen dosage was slowly increased in the following months at the outpatient clinic to manage the spasticity, without further complications.

### Case 2

A 24-year-old man with a neurodegenerative disorder associated with severe bilateral spasticity was hospitalized for wrist arthrodesis of both wrists because of flexion contractures caused by spasticity. At the time of surgery, his baclofen pump was set at 672 mcg/d. Perioperatively, he received multiple doses of fentanyl, and after surgery, he received 3 mg of piritramide i.v. and 7 mg of piritramide subcutaneously at the PACU. In addition to paracetamol and diclofenac, 100 mg tramadol (suppository form) was prescribed twice daily, which the patient first received the evening after surgery at the ward. According to the patient's mother, he was less alert than usual the days after surgery. Nevertheless, the patient was discharged home 2 days after surgery.

Two days later, the patient presented with a fever and tachypnea to the emergency room, and a urosepsis was diagnosed. The patient was admitted to the hospital and treated with antibiotics. He also required supplemental oxygen. Several hours later, the patient had an altered consciousness and only reacted to a painful stimulus. As he remained less alert, tramadol was discontinued. In the following days, his consciousness normalized, but he still had increased respiratory effort, with episodes of tachypnea and apneas. Additional cardiopulmonary examinations showed no cause for the respiratory symptoms. Six days after tramadol was discontinued, the baclofen was reduced to 550 mcg/d upon suspicion of overdose, after which the patient improved clinically, and his respiratory symptoms disappeared. A day later, the patient was discharged.

### Case 3

A 14-year-old boy with neurodegeneration with brain iron accumulation was hospitalized for laparoscopic gastrostomy placement. At the time of surgery, his ITB dosage was 364.5 mcg/d, including flexible programming with three boluses of 75 mcg/bolus per day. Perioperatively, he received remifentanil and, near the end of the procedure, 5 mg piritramide to achieve adequate pain relief. These opioids were deliberately chosen because of their short duration of action (remifentanil; half-life: 3–10 minutes, peak effect: 1–2 minutes) and faster peak effect (piritramide; half-life: 4–12 hours, peak effect: 10 minutes) compared to morphine (half-life: 2–3 hours, peak effect: 20 minutes). 24 At the end of the procedure, there was no spontaneous breathing, and a bolus of 40 mcg naloxone was given twice. After this, adequate ventilation ensued, and the patient was successfully extubated. The patient recovered slowly after anesthesia but was stable, could easily be awakened, and was reacting adequately. Therefore, he was transferred to the regular ward. Four hours after surgery, he was given his regular medication, which consisted of 5 mg baclofen orally, 1. 5 mg clonazepam, and 600 mg gabapentin. Six and half hours after surgery, monitoring of vital functions was started as the patient became less responsive. Another hour later, he was unresponsive to a painful stimulus, had small pupils, and required supplemental oxygen by a nonrebreathing mask. As he stayed unresponsive, with a Glasgow Coma Scale (GCS) of E1M4V1, he received another two boluses of naloxone. Immediate recovery of consciousness occurred (GCS: E3M6V4). Two hours later, another episode of unresponsiveness (GCS: E1M4V1) occurred, and 0.2 mg naloxone was administered. After this dose, consciousness recovered completely, and a continuous infusion of 0.13 mg/h naloxone was started. A few hours later, the Naloxone could be stopped, and the patient recovered to his preoperative condition.

### Case 4

A 15-year-old girl with bilateral spastic cerebral palsy and epilepsy was hospitalized for laparoscopic jejunostomy placement because of feeding problems. Her ITB dose was 507 mcg/d, including a flexible program with four boluses (of 30, 105, 80, and 105 mcg) a day. ITB therapy was continued during and after surgery. Perioperatively, she received 4 mg morphine i.v. and paracetamol i.v. to achieve adequate pain relief. After surgery, she was monitored closely at the pediatric intensive care unit because of the concomitant use of ITB and morphine; overnight, she remained respiratory stable without ventilatory support. The following day, an i.v. morphine infusion was started (5 mcg/kg/h) because of pain. In the afternoon, she was transferred to the pediatric ward. There, she developed apneas and hypoventilation (lowest respiration rate: 7 breaths/min). Oxygen saturation occasionally dropped below 80% with spontaneous recovery. The cause of her apneas was attributed to morphine combined with ITB, and her severe obstructive sleep apnea syndrome (OSAS) with hypoventilation. Two days after the morphine was started, the morphine was switched to oral tramadol (35 mg, four times a day), after which the frequency of apneas decreased. Two days later, the tramadol was discontinued, and in the following days, the frequency of apneas was further decreased to her preoperative, OSAS-associated status.

### Literature Overview

A total of 384 records were identified, and their abstracts were reviewed. Subsequently, potentially relevant articles underwent full-text screening, revealing two articles detailing a total of nine unique cases of severe symptoms of CNS-depressant overdose after concomitant use of opioids and ITB ( Table 3 ). 21 25 These were all pediatric cases, with a mean age of 10.8 years. Five children were comatose, 25 seven needed supplementary oxygen, 21 25 and four had respiratory depression. 21 Three children were treated with naloxone. 21 Formal causality assessment, such as for example, the DIPS score was not reported for any of these cases.

**Table 3:** Literature overview of cases with severe symptoms of CNS-depressant overdose after the concomitant use of ITB and opioids

Ref	Weight (kg)	Age (y)	Diagnoses	Intervention	Baclofen	Intraoperative CNS depressants	Postoperative CNS depressants	Outcome
Anderson et al, 2002 25	22.5	9	CP	Baclofen pump implantation	ITB start: end of surgery Concentration: 2,000 µL/mL Infusion rate: 100 µg/d Possible max inadvertent bolus during surgery: 460 µg	IV sufentanil or fentanyl	IV Morphine	GCS: 6 1.9 h after start of baclofen pump dead space purge; 1.5 h after morphine administration. Needed supplementary oxygen (OS: 94%). Full recovery after 14 h.
	13.6	7	CP	Baclofen pump implantation	ITB start: end of surgery Concentration: 1,000 µL/mL Infusion rate: 100–125 µg/d Possible max inadvertent bolus during surgery: 230 µg	IV sufentanil or fentanyl	IV morphine, IV dimenhydrinate 1.1 mg/kg	GCS: 7 3.8 h after start of baclofen pump dead space purge; 3.6 h after morphine administration; 50 min after dimenhydrinate administration. Full recovery after 1.8 h.
	15.0	5	CP	Baclofen pump implantation	ITB start: end of surgery Concentration: 1,000 µL/mL Infusion rate: 125 µg/d Possible max inadvertent bolus during surgery: 230 µg	IV sufentanil or fentanyl	IV morphine	GCS: 6 1.8 h after start of baclofen pump dead space purge; 1.5 h after morphine administration. Baclofen infusion rate reduced to 100 µg/d. Full recovery after 15 min.
	29.5	12	CP	Baclofen pump implantation	ITB start: end of surgery Concentration: 1,000 µL/mL Infusion rate: 100–125 µg/d Possible max inadvertent bolus during surgery: 230 µg	IV sufentanil or fentanyl	IV morphine	GCS: 6 2 h after start of baclofen pump dead space purge; 1.7 h after morphine administration. Needed supplementary oxygen. Baclofen infusion was stopped. Full recovery after 7.5 h.
	20.4	7	CP	Baclofen pump implantation	ITB start: end of surgery Concentration: 1,000 µL/mL Infusion rate: 100 µg/d. Possible max inadvertent bolus during surgery: 230 µg	IV morphine		GCS: 7 upon arrival in PACU and 1.5 h after intra-OP morphine administration. Woke up after 2 h for 30 min. Again GCS 7. Needed supplementary oxygen (OS: 97% on 4 L/min oxygen). Full recovery after 2 h.
Rizzo et al, 2019 21	30.7 ± 9.6 a	13		Posterior spinal fusion	Stable doses ITB for at least 6 mo	Fentanyl 2 µg/kg, IV ketamine 0.1 mg/kg, IT morphine 20 µg/kg	Bolus morphine 2 mg/mL + ketamine 2 mg/mL (10.60 ± 17.55 µg/kg/h a )	Respiratory depression on postoperative day 0 and 1. Needed supplementary oxygen.
	30.7 ± 9.6 a	17		Posterior spinal fusion	Stable doses ITB for at least 6 mo	CI sufentanil 2 µg/kg/h, bolus sufentanil (range 0.1–0.2 µg/kg), IV ketamine 0.1 mg/kg	Bolus morphine 2 mg/mL + ketamine 2 mg/mL (10.60 ± 17.55 µg/kg/h a )	Respiratory depression on postoperative day 0 and 1, naloxone for respiratory depression treatment, needed supplementary oxygen.
	30.7 ± 9.6 a	13		Posterior spinal fusion	Stable doses ITB for at least 6 mo	CI sufentanil 2 µg/kg/h, bolus sufentanil (range 0.1–0.2 µg/kg), IV ketamine 0.1 mg/kg	Bolus morphine 2 mg/mL + ketamine 2 mg/mL (10.60 ± 17.55 µg/kg/h a )	Respiratory depression on postoperative day 0, 1, and 2, naloxone for respiratory depression treatment, needed supplementary oxygen.
	30.7 ± 9.6 a	15		Posterior spinal fusion	Stable doses ITB for at least 6 mo	CI sufentanil 2 µg/kg/h, bolus sufentanil (range 0.1–0.2 µg/kg), IV ketamine 0.1 mg/kg, IT morphine 20 µg/kg	Bolus morphine 2 mg/mL + ketamine 2 mg/mL (10.60 ± 17.55 µg/kg/h a )	Respiratory depression on postoperative day 0, naloxone for respiratory depression treatment, needed supplementary oxygen.

Abbreviations: CI, continues infusion; CP, cerebral palsy; GCS, Glasgow Coma Scale; IT, intrathecal; ITB, intrathecal baclofen; IV, intravenous; OS, oxygen saturation; PACU, postanesthesia care unit.

a The mean weight/opioid consumption of the 13 children on ITB therapy included in this study.

## Discussion

This report describes four cases detailing the symptoms of CNS-depressant overdose after concomitant use of opioids and ITB within our hospital settings, alongside an overview of nine previously reported cases relating to this interaction in the literature. To our knowledge, including our cases, there are now 13 detailed described cases in the literature with a probable interaction between opiates and ITB resulting in decreased consciousness and/or respiratory depression. Our report is the first to assess the likelihood of this drug interaction using the DIPS. The cases we describe showed decreased consciousness (cases 1, 2, and 3) and respiratory depression with the need for supplemental oxygen (cases 2, 3, and 4) after the coadministration of opioids and ITB. According to the DIPS, there was a possible cause relationship between the opioid-ITB interaction in case 2 and a probable cause relationship in the other cases (cases 1, 3, and 4) and the observed symptoms. Discontinuing opioids improved the consciousness state of the patient in case 2 and improved the respiratory problems of the patient in case 4. Lowering the baclofen dose led to a gradual recovery of the consciousness state of the patient in case 1 and improved the respiratory issues of the patient in the second case. In the third case, naloxone, an opioid antagonist, ultimately abolished the symptoms. The initially observed partial effect of naloxone, characterized by symptom recurrence a few hours after each naloxone dose, can be attributed to the pharmacokinetic interplay between naloxone and the opioids used. Naloxone's efficacy in reversing opioid effects depends on various factors, including opioid dose, residual opioid concentration, potency, and half-life. 26 Given naloxone's relatively short half-life (60–90 minutes 24 ), its duration of action may be surpassed by certain opioids, such as piritramide (half-life 4–12 hours) in this case. Consequently, repeated naloxone administrations or even continuous infusion and respiratory monitoring are essential. The disappearance of CNS depression symptoms upon initiating a continuous infusion is supportive of opioid intoxication as a contributing factor to the symptoms in the third case.

Studies investigating the potentially life-threatening opioids–ITB interaction are scarce, primarily because adverse events are often described without explicit reference to the use of other medications and modes of administration. Anderson et al reported postoperative coma in five out of nine patients after ITB pump implantation. 25 Although they suspected an unintended bolus of ITB as the leading cause of the coma, they also doubted the involvement of baclofen–opioid interaction as the children with postoperative coma had received higher doses of intravenous morphine compared with those without ( Table 3 ). Rizzo et al reported respiratory depression in four out of thirteen children on ITB therapy after major surgery. 21 Those four children received a combination of an opiate with ketamine during surgery, whereas none of the other children on ITB therapy received this combination intraoperatively. Desaturation (oxygen saturation <92%) and somnolence were also more common in children on ITB therapy than in children without ITB therapy who underwent the same surgery (38.5 vs. 5.9% and 61.5 vs. 17.6%, respectively). According to the authors, these results suggest that children on ITB therapy are at a greater risk of developing respiratory depression and oversedation with concomitant opiate use.

Three large retrospective studies assessed the risk of (symptoms of) opioid overdose with concurrent use of opiates and SMRs in the United States. All three studies showed an increased risk of opioid overdose with the concurrent use of opiates and SMR (hazard ratio [HZ]: 1.21 18 ), particularly with baclofen (HZ: 1.83, 18 odds ratio: 1.56, 20 HZ: 2.52 19 ). These studies were conducted in adults only, some of whom were long-term opiate users, and unfortunately, none of these studies mentioned the route of drug administration (oral, intravenous, and intrathecal).

The interaction between opioids and baclofen in the CNS is complex and not yet fully understood. Although the mechanisms of action of baclofen and opioids are generally understood, it is not entirely clear how they may potentiate each other. Baclofen acts as a GABA _B_ receptor agonist, and these receptors are predominantly localized on neurons in the CNS. 6 27 28 By binding pre- and postsynaptically, baclofen inhibits neuronal transmission onto and into lower motor neurons, manifesting its muscle relaxant effect. 28 29 Opioids act by binding to opioid receptors, mainly μ-, κ-, and δ-receptors and are primarily active in the CNS. 16 30 Activation of these receptors on both the presynaptic and postsynaptic sides reduces neuronal activity and exerts its analgesic effect. 30 It is assumed that the opioid–baclofen interaction occurs because of the CNS-depressant properties of both drugs, which may potentiate each other's CNS-depressant effect. 18 19 29 31 Evidence for this assumption can be found in previous studies showing that combined activation of opioid and GABA _B_ receptors leads to enhanced analgesia. 32 33 34 The dorsal horn of the spinal cord and a supraspinal location are considered possible sites for the opioid–baclofen interaction because both GABA _B_ receptors and opioid receptors are found at the same terminals of primary afferent nociceptors. 21 32 33

It is important to realize that the concentrations of baclofen and opioids in the CNS are unevenly distributed. The drug concentrations in the CSF, brain interstitial fluid, and brain regions are unequal. 35 36 37 Moreover, there is a significant reduction in the concentration of baclofen in the CSF cranially along the spinal canal from the insertion site. 38 39 In addition, the rate at which different opioids pass the BBB and are subsequently eliminated depends on their lipophilicity, with morphine passing and being eliminated slowly, while fentanyl and piritramide pass the BBB fast. 17 40 Depending on the specific opioid and the method of administration (e.g. bolus and continuous) concurrent with ITB, the duration of their simultaneous presence in the CNS varies, influencing the potential for mutual reinforcement. It should also be remembered that overdosing on one of those drugs alone can result in severe complications as well.

Our experiences with this interaction have prompted protocol modifications in our centers. When implanting a baclofen pump, our initial choice for analgesia is remifentanil due to its ultrashort duration of action. Fentanyl and sufentanil are also viable options. To control pain postoperatively, it is advisable to minimize the concurrent use of baclofen and opioids. In many cases, adding clonidine to a multimodal pain management approach effectively alleviates pain. Ketamine can also be considered, but be aware that ketamine can prolong the duration of action of muscle relaxants. Local injection of an analgesic (e.g., bupivacaine) or a peripheral nerve blocks can also reduce the need for opioids. If opioids are still deemed necessary, administration of opioids, preferably with a short duration of action, should be closely monitored in the PACU until a stable condition is achieved. This is because there is still an increased and unpredictable risk of respiratory depression and excessive sedation when baclofen and opioids are used concurrently.

In the event of a suspected or confirmed interaction, we recommend temporarily stopping ITB administration or reducing the dose by programming the baclofen pump accordingly. Opioids could be antagonized; however, it should be kept in mind that symptoms of CNS depression may recur over time, potentially requiring repeated doses of naloxone for sustained reversal. Furthermore, parents have a letter explicitly addressing this specific interaction they can hand to (pediatric) anesthesiologists or (pediatric) physicians during each preoperative screening and hospitalization.

In conclusion, with this report, we raise awareness of the risks of concomitant use of baclofen, specifically intrathecally administered and opioids in children and young adults. We provide suggestions for the prevention of interaction and its clinical management. The concomitant use of ITB and opioids may enhance the risk of symptoms of CNS-depressant overdose. Therefore, simultaneous use of both drugs should be a deliberate decision, and clinicians should be aware of the possible consequences of an interaction. In these cases, it is strongly recommended to closely monitor these patients' vital functions to early detect interaction symptoms, such as respiratory depression and decreased consciousness.
